# Gender- and Age-Associated Differences in Bone Marrow Adipose Tissue and Bone Marrow Fat Unsaturation Throughout the Skeleton, Quantified Using Chemical Shift Encoding-Based Water–Fat MRI

**DOI:** 10.3389/fendo.2022.815835

**Published:** 2022-04-27

**Authors:** Kerensa M. Beekman, Martine Regenboog, Aart J. Nederveen, Nathalie Bravenboer, Martin den Heijer, Peter H. Bisschop, Carla E. Hollak, Erik M. Akkerman, Mario Maas

**Affiliations:** ^1^ Department of Radiology and Nuclear Medicine, Amsterdam University Medical Centers, Amsterdam Movement Sciences, University of Amsterdam, Amsterdam, Netherlands; ^2^ Department of Endocrinology, Amsterdam University Medical Centers, Amsterdam Movement Sciences, Vrije Universiteit University, Amsterdam, Netherlands; ^3^ Department of Endocrinology and Metabolism, Amsterdam University Medical Centers, Amsterdam Movement Sciences, University of Amsterdam, Amsterdam, Netherlands; ^4^ Department of Clinical Chemistry, Research Laboratory Bone and Calcium Metabolism, Amsterdam University Medical Centers, Amsterdam Movement Sciences, Vrije Universiteit University, Amsterdam, Netherlands; ^5^ Department of Internal Medicine, Leiden University Medical Center, Leiden, Netherlands

**Keywords:** bone marrow adipose tissue, bone marrow fat unsaturation, bone marrow adipose tissue distribution, healthy subjects, water–fat MR imaging

## Abstract

Bone marrow adipose tissue (BMAT) is a dynamic tissue which is associated with osteoporosis, bone metastasis, and primary bone tumors. The aim of this study is to determine region-specific variations and age- and gender-specific differences in BMAT and BMAT composition in healthy subjects. In this cross-sectional study, we included 40 healthy subjects (26 male: mean age 49 years, range 22–75 years; 14 female: mean age 50 years, range 29–71) and determined the bone marrow signal fat fraction and bone marrow unsaturation in the spine (C3-L5), pelvis, femora, and tibiae using chemical shift encoding-based water–fat imaging (WFI) with multiple gradient echoes (mGRE). Regions of interest covered the individual vertebral bodies, pelvis and proximal epimetaphysis, diaphysis, and distal epimetaphysis of the femur and tibia. The spinal fat fraction increased from cervical to lumbar vertebral bodies (mean fat fraction ( ± SD or (IQR): cervical spine 0.37 ± 0.1; thoracic spine 0.41 ± 0.08. lumbar spine 0.46 ± 0.01; p < 0.001). The femoral fat fraction increased from proximal to distal (proximal 0.78 ± 0.09; diaphysis 0.86 (0.15); distal 0.93 ± 0.02; p < 0.001), while within the tibia the fat fraction decreased from proximal to distal (proximal 0.92 ± 0.01; diaphysis 0.91 (0.02); distal 0.90 ± 0.01; p < 0.001). In female subjects, age was associated with fat fraction in the spine, pelvis, and proximal femur (ρ = 0.88 p < 0.001; ρ = 0.87 p < 0.001; ρ = 0.63 p = 0.02; ρ = 0.74 p = 0.002, respectively), while in male subjects age was only associated with spinal fat fraction (ρ = 0.40 p = 0.04). Fat fraction and unsaturation were negatively associated within the spine (r = -0.40 p = 0.01), while in the extremities fat fraction and unsaturation were positively associated (distal femur: r = 0.42 p = 0.01; proximal tibia: r = 0.47, p = 0.002; distal tibia: r = 0.35 p = 0.03), both independent of age and gender. In conclusion, we confirm the distinct, age- and gender-dependent, distribution of BMAT throughout the human skeleton and we show that, contradicting previous animal studies, bone marrow unsaturation in human subjects is highest within the axial skeleton compared to the appendicular skeleton. Furthermore, we show that BMAT unsaturation was negatively correlated with BMAT within the spine, while in the appendicular skeleton, BMAT and BMAT unsaturation were positively associated.

## Introduction

Bone marrow adipose tissue (BMAT) is associated with different diseases like osteoporosis (1–3) and primary bone malignancies (4); furthermore, the bone marrow is a frequent metastatic site. BMAT is associated with bone metabolism (5, 6) and serves as a lipid source for proliferation and infiltration of malignant cells (7, 8), and tumor cells can affect the secretion of free fatty acids by bone marrow adipocytes (9). Furthermore, lower BMAT unsaturation and higher BMAT saturation appear to be associated with fractures (10).

Traditionally, the bone marrow is divided into red (or hematopoietic) and yellow (or fatty) marrow (11). After birth, conversion of the red marrow into yellow marrow starts in the appendicular skeleton in a centripetal way (12), and BMAT increases by approximately 6%–7.5% per decade (13–16). In situations of increased hematological demands, reconversion of yellow marrow to red marrow may occur. Furthermore, there are gender-associated differences in BMAT (13–15, 17).

Little is known about the region-specific variation bone marrow fatty acid composition in humans. Animal studies suggest that there are two distinct types of BMAT: regulated and constitutive BMAT (rBMAT and cBMAT) (18). In animals, rBMAT contains more saturated fatty acids, is located proximally within long bones, develops throughout life, and responds to BMAT-inducing stimuli. cBMAT contains more unsaturated fatty acids, is located distally in long bones, develops in early life, and does not respond to BMAT-inducing stimuli (18).

The gold standard for BMAT and BMAT fatty acid composition is single-voxel proton magnetic resonance spectroscopy (^1^H-MRS) (19). However, ^1^H-MRS availability is limited and the sampling area within the skeleton small, due to long acquisition times. Chemical shift encoding-based water–fat imaging (WFI) is widely used for quantification of BMAT (19). WFI with multiple gradient echoes (WFI-mGRE) is able to quantify both bone marrow fat fraction and BMAT unsaturation from the same MRI images, with fast acquisition times. Furthermore, WFI-mGRE shows good agreement with ^1^H-MRS (20–24).

Insight in the normal variation of BMAT and BMAT fatty acid composition throughout the skeleton is necessary to further evaluate the interaction between BMAT, bone metabolism, and skeletal malignancies. Therefore, we aimed to determine region-specific variation and age- and gender-specific differences in BMAT and BMAT composition using WFI-mGRE, in healthy subjects.

## Methods

### Subjects

In this cross-sectional study, we quantified the bone marrow signal fat fraction in 40 healthy subjects within the spine (C3-L5), pelvis, femora, and tibia. All subjects were recruited as part of a different study (25) (trial nr. NTR5056). The main inclusion criterion was age >18 years, and subjects with a history of bone marrow disease were excluded. Subjects had no hematologic or metabolic disorders. One subject used vitamin D supplementation, and two subjects used oral contraceptives.

We included 26 male subjects and 14 female subjects. The mean age was 49 years for male subjects (range 22–75 years) and 50 years (range 29–71) for the female subjects (p = 0.84). Seven out of the 14 female subjects were postmenopausal. Subject characteristics are shown in **Table 1**. Male subjects were taller compared to female subjects (mean height: male 182 ± 8 cm, female 170 ± 7 cm; p < 0.001) and were heavier (mean weight: male 84 ± 13 kg; female 68 ± 12 kg; p < 0.001). BMI was not significantly different between male and female subjects (mean BMI male: 25.5 ± 0.6; female: 23.6 ± 0.5; p = 0.16).

**Table 1:** Subject characteristics, mean ± SD.

	Male	Female
N	26	14
Age (years)	49 (range 22–75)	50 (range 29–71)
Postmenopausal		7 out of 14
Height (cm)	182 ± 8	170 ± 7
Weight (kg)	84 ± 14	68 ± 12
BMI (kg/m^2^)	25.5 ± 3.4	23.6 ± 4.4
Hb	9.3 ± 0.6	8.3 ± 0.5

Scans were acquired between July 2014 and August 2015 at the Amsterdam University Medical Centers/University of Amsterdam. The local ethics committee of the Amsterdam University Medical Centers/University of Amsterdam approved the protocol, and all subjects gave their written informed consent. This study was carried out in compliance with the World Medical Association Declaration of Helsinki—Ethical Principles for Medical Research Involving Human Subjects.

### Image Acquisition

From the previous study with trial number NTR5056, we used a coronal whole-body and a sagittal whole-spine data set. All images were acquired on a 1.5-T MRI (Siemens Avanto, Siemens AG, Erlangen, Germany). All patients were placed in the scanner in supine position. Both datasets consisted of magnitude and phase images, enabling complex valued analysis. Both datasets were acquired using a standard 2D multi-echo spoiled gradient echo sequence, with 12 echoes, TE = 0.99–16.5 ms and an echo spacing of 1.41 ms. Slice thickness was 7.5 mm, with a 7.5-mm gap. The flip angle was 20°, and the acquisition matrix was 256 × 96.

The whole-body data set was acquired with 8 to 9 stations of 15 to 21 slices, a repetition time of 301 ms, and a field of view of 250 × 500 mm. Acquisition time is 17.8 s per station (breath-hold for the thorax and abdominal stations). The spine data set was acquired with 3 to 5 stations of 15 slices, a repetition time of 333 ms, and a field of view of 280 × 280 mm. Acquisition time is 19.7 s per station.

### Region of Interest

Regions of interest (ROIs) were always drawn within the data of one station, since phase information from different stations does not match. The fitted parameters from different ROIs were averaged when necessary. In the spine, usually one mid-sagittal ROI per vertebral body was drawn. For the pelvis, several ROIs in 3 slices where the pelvis bone marrow was manifestly visible, both right and left, were drawn. For both the femora and tibiae, right as well as left, three ROIs were drawn: the proximal epimetaphysis, the diaphysis, and the distal epimetaphysis, again in the image where the bone marrow was most clearly visible. Examples of the ROIs covering the spine, pelvis, femora, and tibiae are shown in **Supplemental Figure 1**.

### Analysis of the ROIs

The multi-echo data were fitted to a fat–water signal function, based on the fat characterization model by (26).


$$S(TE)={\text{e}}^{i(\varphi_{0}+\omega_{0}TE)}\cdot S_{0}\cdot \left\{(1-S_{FF})\;{\text{e}}^{-TE/T_{2,W}^{*}}+S_{FF}\;{\text{e}}^{-TE/T_{2,f}^{*}}\sum_{n-1}^{9}{\text{e}}^{-i\;\omega_{n}TE}v_{n}(CL,dnb,nmidb)\right\}$$

The model includes nine fat peaks, each with its own frequency with respect to the water peak, *ωn*, and weighting factor, *νn*, which in its turn depends on the average fatty acid chain length (*CL*), the number of double bonds per fat molecule (*ndb*), and the number of direct double-bond neighbor pairs (methylene-interrupted double bonds, *nmidb*). The weighting factors were scaled so that their sum equaled one. It turned out that our data were not sufficient enough to fit all three parameters reliably, so we fixed the *CL* to 17.45, being the average chain length in human fat (20), thus leaving *ndb* and *nmidb* as free parameters to characterize the fat composition.

**Figure 1:**
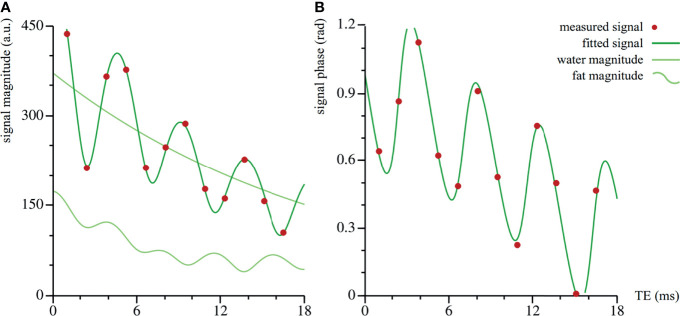
Dataset and corresponding fit of vertebra Th11 of volunteer #20. The fit is performed on the complex valued data: the magnitude (arbitrary units) in **(A)**, the phase (radians) in **(B)**. The amplitude in the total signal magnitude is larger than in the fat component only, because of the interference of the fat and water signals.

Further free parameters are as follows: *φ*0, the excitation phase offset, *ω*0, the frequency contribution of *B*0 inhomogeneities, *S*0, the signal magnitude at t = 0, *S_FF_*, the signal fat fraction, *T*2,w*, the water relaxation time, and *T*2,f*, the fat relaxation time, which is taken to be equal for all fat peaks. Our data set did not contain information to correct for differences in *T*1, which would require additional acquisitions, therefore measuring the signal fat fraction, rather than the actual proton density fat fraction. In the tibiae and femora, a pixel-by-pixel fit could be performed. In the spine and pelvis, we first estimated an averaged signal over the ROI. This estimation was done for the signal magnitude and phase separately. **Figure 1** shows an example of a dataset and the corresponding fit.

### Statistical Analysis

The statistical analysis was performed with IBM SPSS Statistics for Windows (version 26; SPSS Inc., Chicago, IL, USA). Graphs were created using GraphPad Prism (Version 8.2.1 for Windows, GraphPad Software, La Jolla, CA, USA). The mean and standard deviation (reported as “ ± SD”) or the median and interquartile ranges (reported as “(IQR)”) are reported, depending on the distribution. To compare male and female subject characteristics, we used Student’s t-test or the Mann–Whitney U test, depending on the distribution of the data. Within-subject differences in BMAT distribution and unsaturation linear mixed models (LMM) were used. A covariance structure was chosen based on Akaike’s information criterion (AIC) and Schwarz’s Bayesian criterion (BIC). We used Pearson (r) and Spearman’s (ρ) tests to determine correlations between variables depending on the distribution of the data. To estimate the effects of age, gender, and unsaturation (*ndb*) and their interactions on BMAT (fat fraction), multiple linear regression models were used. BMAT was included as the outcome variable, and the age and number of double bonds and their interactions were included as predictors. In case assumptions were violated (normally distributed residuals, equal variances), the outcome variable was rank transformed. All statistical tests were two-sided, and a p-value of 0.05 was considered significant. For the *post-hoc* analysis of the LMM, a Bonferroni correction was applied, making p < 0.008 significant for the comparison between the spine, pelvis, femur, and tibia and p < 0.016 for the comparisons within the spine, femur, and tibia.

## Results

### Distribution of Bone Marrow Adipose Tissue and Bone Marrow Fat Composition

**Figure 2:**
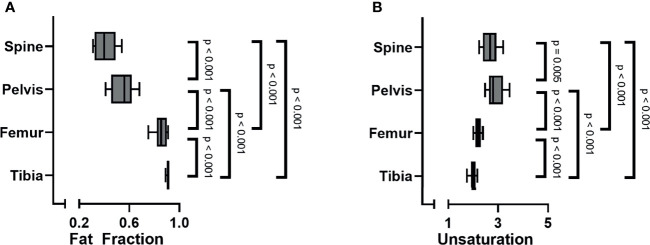
**(A)** Bone marrow fat fraction increased from cranial to caudal (linear mixed model (LMM): p < 0.001). **(B)** Unsaturation (number of double bonds, *ndb*) was highest within the pelvis, followed by the spine, and lowest within the tibia (LMM: p < 0.001).

**Figure 3:**
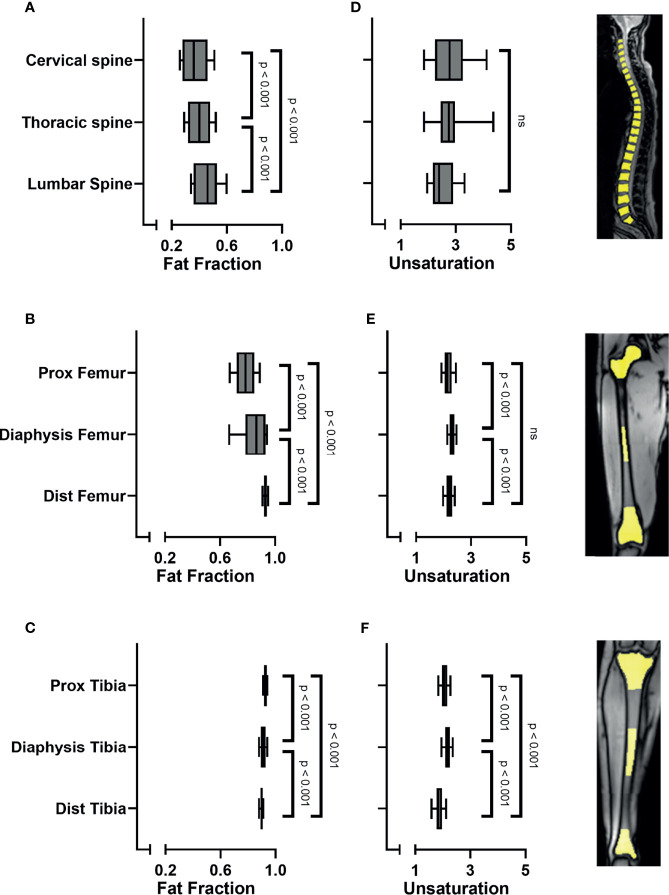
**(A)** Bone marrow fat fraction increased from cranial to caudal within the spine (linear mixed model (LMM): p < 0.001). **(B)** Fat fraction increased in the femur from proximal to distal (LMM: p < 0.001). **(C)** Fat fraction decreased in the tibia from proximal to distal (LMM: p < 0.001). **(D)** Unsaturation (number of double bonds, *ndb*) was similar when comparing the cervical spine, thoracic spine, and the lumbar spine (LMM: p = 0.09; ns). **(E)** Unsaturation was highest within the diaphysis of the femur (p < 0.001) and **(F)** within the diaphysis of the tibia (LMM: p < 0.001), compared to the proximal and distal sites. Images on the right side show representative images of ROI placement in the vertebral bodies of the spine, in the proximal epimetaphysis, the diaphysis and the distal epimetaphysis of the femora and tibiae. ns, non-significant.

When analyzing all subjects collectively, the bone marrow fat fraction increased from cranial to caudal, with the spine having the lowest fat fraction and the tibia the highest (spine 0.41 ± 0.09; pelvis 0.56 ± 0.1; femur 0.86 (0.08); tibia 0.91 (0.02) p < 0.001; **Figure 2A**). Similarly, within the spine the fat fraction increased from cranial to caudal (**Figure 3A**), with the cervical spine having a significantly lower fat fraction compared to the thoracic and lumbar spine (cervical spine 0.37 ± 0.1; thoracic spine 0.41 ± 0.08; lumbar spine 0.46 ± 0.01; p < 0.001). Within the femur, the fat fraction increased from proximal to distal (proximal 0.78 ± 0.09; diaphysis 0.86 (0.15); distal 0.93 ± 0.02; p < 0.001; **Figure 3B**), while within the tibia the fat fraction showed a small, but significant decrease from proximal to distal (proximal 0.92 ± 0.01; diaphysis 0.91 (0.02); distal 0.90 ± 0.01; p < 0.001; **Figure 3C**). Throughout our data, the resulting fitted values of methylene-interrupted double bonds *(nmidb)* were close to zero. Consequently, we cannot draw any conclusions from this parameter, and we only present the values for *ndb* as a measure for bone marrow fat unsaturation. Unsaturation was highest in the pelvis, second highest in the spine, and lowest in the tibia (spine 2.71 ± 0.35; pelvis 2.82 (0.56); femur 2.22 ± 0.14; tibia 2.03 ± 0.16; p < 0.001; **Figure 2B**). Unsaturation was not significantly different when comparing the cervical spine, the thoracic spine, and the lumbar spine (cervical spine 2.89 ± 1.04; thoracic spine 2.74 ± 0.50; lumbar spine 2.56 ± 0.53, p = 0.13); **Figure 3D**). Within both the femur and the tibia, unsaturation was highest within the diaphysis (femur: proximal 2.16 ± 0.19; diaphysis 2.31 ± 0.13; distal 2.2 ± 0.15; p < 0.001; **Figure 3E** and tibia: proximal 2.06 ± 0.16; diaphysis 2.17 ± 0.15; distal 1.85 ± 0.20; p < 0.001; **Figure 3F**) . No significant correlations were found between unsaturation and age, neither in female nor in male subjects; results are shown in **Supplemental Figure 2**.

### Age- and Gender-Related Differences in Bone Marrow Adipose Tissue

**Figure 4:**
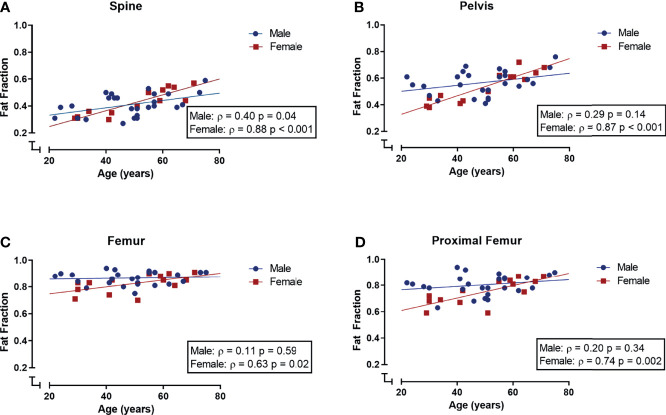
Correlations (Spearman’s rank correlation coefficient) between age and bone marrow fat fraction (*S_FF_*) in male (blue) and female (red) subjects. **(A)** Spinal fat fraction and age were positively correlated in both male and female subjects. **(B–D)** Within the pelvis, femur, and proximal femur fat fraction and age were positively correlated in female subjects but not in male subjects.

When analyzing all subjects collectively, the bone marrow fat fraction increased with age in the spine and pelvis (spine: r = 0.64 and p < 0.001; pelvis: r = 0.56 and p < 0.001; data not shown). Within the extremities, the fat fraction of the femur tended to correlate with age, based on a positive correlation between the fat fraction of the proximal femur and age (femur: ρ = 0.27, p = 0.09; proximal femur: r = 0.41, p = 0.01; tibia ρ = - 0.23, p = 0.16; data not shown). Within female subjects, bone marrow fat fraction and age were positively correlated within the spine, pelvis, femur, and proximal femur (spine: ρ = 0.88, p < 0.001; pelvis: ρ 0.87, p < 0.001; femur: ρ = 0.63, p = 0.02; proximal femur: ρ = 0.74, p = 0.002; **Figures 4A–D**), while in male subjects fat fraction and age were only correlated within the spine, and not within the pelvis, femur, proximal femur, and tibia (spine: ρ = 0.40, p = 0.04; pelvis: ρ = 0.29, p = 0.14; femur: ρ = 0.11, p = 0.59; proximal femur: ρ = 0.20, p = 0.34 (**Figures 4A–D**); tibia ρ = -0.29, p = 0.16; data not shown). Multiple linear regression models were applied to estimate the effect of age, gender, and their interaction on the bone marrow fat fraction. Within the spine, only age was a significant predictor of fat fraction (p < 0.001, R^2^ = 0.40). Within the pelvis, age, gender, and their interaction term were significant predictors of the fat fraction (age: p < 0.001; gender p = 0.01; age*gender p = 0.02; R^2^ = 0.41). Within the femur, only gender was a significant predictor of the fat fraction (p = 0.025; R^2^ 0.19); no significant interaction between age and gender was found within the total femur. Within the proximal femur, age, gender, and the interaction term were significant predictors of the fat fraction (age: p = 0.001; gender p = 0.01; age*gender p = 0.05; R^2^ = 0.34). Regression lines are shown in **Figure 4**.

### The Association Between BMAT Amount and BMAT Composition

**Figure 5:**
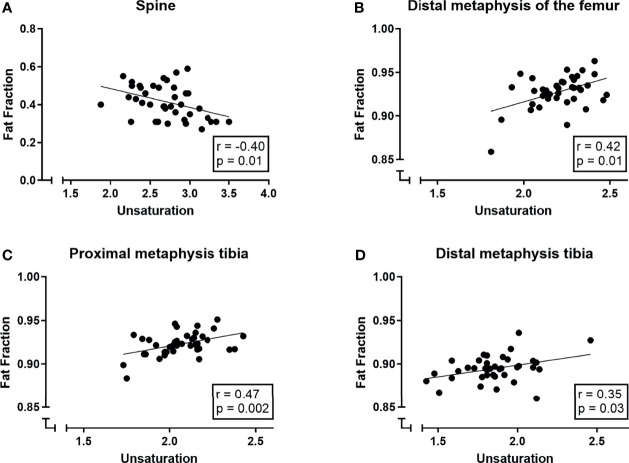
Correlations (Pearson correlation coefficient) between bone marrow signal fat fractions (*S_FF_*) and unsaturation (number of double bonds *ndb*) in **(A)** the spine, **(B)** the distal femur, **(C)** the proximal tibia and **(D)** the distal tibia.

Despite age and gender being significant predictors of fat fraction, we previously showed that there were no significant correlations between age, gender, and unsaturation (Section 3.1; **Supplemental Figure 2**). Therefore, we decided to combine our subjects to evaluate bone marrow fat fractions and unsaturation. Within the spine, there was a negative correlation between bone marrow fat fraction and unsaturation (r = -0.40, p = 0.01; **Figure 5A**). While in the distal femur, the total tibia and in the proximal and distal tibia fat fraction and unsaturation were positively correlated (distal femur: r = 0.42, p = 0.01, **Figure 5B**; total tibia: ρ = 0.52, p = 0.05, data not shown; proximal tibia: r = 0.47 p = 0.002, **Figure 5C**; distal tibia: r = 0.35, p = 0.03 **Figure 5D**). No correlation between fat fraction and unsaturation was found at the other skeletal sites (pelvis: r = 0.16, p = 0.31; femur: ρ = -0.26 p = 0.37; proximal femur: -0.01, p = 0.95; diaphysis of the femur: ρ -0.16, p = 0.58; and the diaphysis of the tibia: r = 0.13, p = 0.43; data not shown).

A multiple linear regression model was used to predict the outcome variable (fat fraction/*S_FF_*) based on unsaturation, age, and gender and their interaction terms. Within the spine, age and unsaturation were significant predictors of the fat fraction (age: p < 0.001; *ndb* p = 0.02; R^2^ of 0.50); gender and interaction terms were not significant predictors for the fat fraction in the spine. In the distal femur, the total tibia, and the proximal and distal tibia, only unsaturation was a significant predictor of the fat fraction while gender, age, or interaction terms were not (*ndb*: distal femur: p = 0.005, R^2^ 0.23; total tibia: p = 0.03, R^2^ 0.19; proximal tibia: p = 0.002, R^2^ 0.24; distal tibia: p = 0.02, R^2^ 0.18).

## Discussion

To our knowledge, we are the first to report both BMAT distribution and BMAT unsaturation, and the association between BMAT and BMAT unsaturation, quantified simultaneously with WFI-mGRE, in a large part of the skeleton in a group of healthy subjects.

We show that BMAT increases from spine to tibia, from the cervical to the lumbar spine, and from proximal to distal in femora, while we show a small but significant decrease within tibia. These patterns are consistent with literature (13–15, 27–31). Furthermore, we confirm differences in the age-associated increase in BMAT between male and female subjects (15, 29, 31). BMAT within the femur and tibia does not increase with age in male subjects, while in female subjects femoral BMAT does increase with age. This could indicate that BMAT in the axial and appendicular skeleton is differently regulated in female and male subjects and suggests a role for sex steroids, as it is known that exogenous estradiol can decrease BMAT (32, 33), and low endogenous testosterone is associated with high BMAT in older men (34). Changes in sex steroids with aging, and especially during menopause in female subjects, could explain these gender differences observed in femoral BMAT. Another explanation for the gender difference at a younger age could be increased hematopoietic demands in premenopausal women due to blood loss during menstrual periods, as BMAT is also potentially linked to erythropoiesis (35).

We show higher BMAT unsaturation within the axial skeleton compared to the appendicular skeleton. Our results are inconsistent with animal studies (18, 36), showing higher unsaturation in areas of yellow bone marrow compared to red bone marrow. However, few studies have quantified BMAT unsaturation in both the axial and appendicular skeleton in human subjects. Our results are consistent with a small study by Badr and coworkers which showed higher unsaturation within the pelvis compared to the proximal femur in young female subjects, quantified by ^1^H-MRS (37). This could imply that BMAT unsaturation in areas red bone marrow in the axial skeleton and yellow bone marrow of the appendicular skeleton might differ between human subjects and these animal models. We showed that BMAT unsaturation was higher within the femur compared to the tibia and that BMAT unsaturation was higher within the diaphysis of the tibia and femur compared to the proximal or distal metaphysis, but we found no gender- or age-associated differences in BMAT unsaturation. Our results are comparable to a study by Bao who reported higher BMAT unsaturation within the distal femur compared to the proximal tibia, and no differences in BMAT unsaturation between young male and female subjects (38). Our results partially differ from the study by Scheller and coworkers in 5 young female subjects, as they report higher BMAT unsaturation, measured using ^1^H-MRS, within the tibia compared to the femur. However, unsaturation was higher within the diaphysis of the tibia and femur, although not significantly in the study by Scheller and coworkers. The difference could be due to the sample size, or age and gender differences, as Scheller and coworkers included only 5 young, female subjects, and we included both male and female subjects with a larger age range (18). Other studies that quantified BMAT unsaturation in proximal and distal skeletal sites in human subjects show conflicting results, possibly due to small sample sizes, and differences in age and gender of the subject (39–41). To the best of our knowledge, there are no other studies quantifying BMAT composition in both the axial and appendicular skeleton in human subjects. Due to heterogeneity in used imaging techniques, in scanner vendors, and in study populations, results from different studies are hard to compare. The negative correlation we found between BMAT and unsaturation in the spine, consistent with a previous study (42), opposed to the positive correlation between BMAT and unsaturation we found within the appendicular skeleton (distal femur, and proximal and distal tibia), could support the existence of different types of BMAT in the axial versus appendicular skeleton, also referred to as regulated and constitutive BMAT, as reviewed by Craft et al. (43), with a distinct fatty acid composition and with different effects on bone metabolism (44–48) and on skeletal metastasis. Another potential explanation for the difference in unsaturation between the axial and appendicular skeleton in healthy subjects could be glucose metabolism as a recent study by Suchacki and coworkers showed that BMAT glucose uptake was higher within the axial skeleton compared to BMAT glucose uptake within long bones (49).

Patients with osteoporosis and subjects with fractures have lower unsaturation and higher saturation of their BMAT compared to healthy subjects or subjects without fractures (10, 42). *In vitro* research has shown that saturated fatty acids can increase osteoclast differentiation, decrease osteoblastic differentiation, and induce a pro-inflammatory response, while unsaturated fatty acids could prevent these effects (50). Furthermore, BMAT could serve as an energy depot for bone metabolism and bone metastasis. Although most tumors depend on glycolysis for their energy supply, β-oxidation of fatty acids can serve as a main source of energy for some types of cancers (9). Tumor cells can stimulate lipolysis and the secretion of free fatty acids by bone marrow adipocytes (51) and overexpress lipid transporters to increase lipid uptake (52–54). Furthermore, multiple enzymes of the desaturase pathway are overexpressed in tumor cells of metastatic prostate cancer and multiple myeloma; however, in the hypoxic environment of the bone marrow the function of the enzyme stearoyl-CoA desaturase is compromised and therefore the synthesis of monounsaturated fatty acids. Under these hypoxic conditions, tumor cells can switch to collecting unsaturated fatty acids from the microenvironment (9), and it could be proposed that, although highly speculative, the increased unsaturated fatty acids we show within the spine and pelvis compared to the femora and tibia might be a part of the explanation why skeletal metastases are preferentially located within the axial skeleton (55). Furthermore, it could be postulated, again highly speculatively, that the gender-associated differences in BMAT within the spine, pelvis, and proximal femur, i.e., areas containing red bone marrow, could be part of the explanation for the observation that female patients are less likely to have skeletal metastasis (56) and more likely to develop osteoporosis. Future research on the interaction between BMAT and bone metabolism or skeletal metastasis should take these differences between the axial and appendicular skeleton into consideration, as results on BMAT acquired from the iliac crest might provide different results compared to BMAT acquired from the proximal femur.

Our study has limitations. First, we did not compare the WFI-mGRE to a reference method, like gas chromatography or ^1^H-MRS. As subjects were included as part of a different study protocol, data of the gradient echo MRI images were analyzed retrospectively. Nevertheless, previous studies have demonstrated good agreement between WFI-mGRE and ^1^H-MRS (20, 22). A second limitation is that we measure the bone marrow fat signal fraction instead of the corrected proton density fat fraction (PDFF). Although we did use a multipeak fat spectrum for the fit and corrected for T2* decay, we did not correct for T1 bias. The relatively short T1 of fat compared to water could cause higher fat fractions in areas where fat is less than water or lower fat fractions when fat is higher than water (57). Therefore, our results within the appendicular skeleton, where fat is much higher than water, likely underestimate the actual PDFF. Fat and water fractions within the spine and pelvis are more balanced, i.e., fat fractions closer to 50%, causing less T1 bias. Therefore, our results of the spine and pelvis are more comparable to the actual PDFF. T1 bias increases with larger flip angles. As we used a flip angle of 20° in our retrospective analysis, T1 bias could be further reduced by using a smaller flip angle in future studies. Last, our sample size is relatively small; therefore, our subanalyses are likely subject to power issues. For example, only 14, both premenopausal and postmenopausal, women were included, which potentially influenced our results. Due to the retrospective nature of our study, it was not powered for these analyses. Therefore, future research should reproduce our data.

To conclude, we show that off-shelf GRE sequences can be used to quantify BMAT and BMAT composition simultaneously, in large parts of the skeleton. We describe the distribution of BMAT and BMAT unsaturation within a group of healthy subjects and report age- and gender-associated differences. Contradicting previous animal studies, we show higher unsaturation within the axial skeleton (i.e., red marrow) compared to the appendicular skeleton (i.e., yellow marrow) and we show opposing correlations between BMAT and BMAT unsaturation when comparing the spine to the femur and tibia; this could support the existence of two distinct types of BMAT within the axial versus appendicular skeleton, potentially with different interactions with its environment. Our results supply a useful ground for future research on the interaction between BMAT and different (patho)physiological processes like bone metabolism and skeletal metastasis.

## Data Availability Statement

The raw data supporting the conclusions of this article will be made available by the authors, without undue reservation.

## Ethics Statement

The studies involving human participants were reviewed and approved by the Ethics Committee of the Amsterdam University Medical Centers/University of Amsterdam. The patients/participants provided their written informed consent to participate in this study.

## Author Contributions

Study design: KB, MR, CH, PB, MM. Study conduct: MR, EA, and CH. Data collection: MR, EA. Data analysis: KB, EA, and AN. Data interpretation: KB, EA, AN, NB, PB, MH, and MM. Drafting of the manuscript: KB, EA and MM. All authors critically revised the manuscript and approved the final version of the manuscript to be submitted.

## Funding

KMB is supported by an Alliance grant provided by Amsterdam University Medical Centers/VU University/University of Amsterdam (2013–01).

## Conflict of Interest

The authors declare that the research was conducted in the absence of any commercial or financial relationships that could be construed as a potential conflict of interest.

## Publisher’s Note

All claims expressed in this article are solely those of the authors and do not necessarily represent those of their affiliated organizations, or those of the publisher, the editors and the reviewers. Any product that may be evaluated in this article, or claim that may be made by its manufacturer, is not guaranteed or endorsed by the publisher.

## Abbreviations

BMAT, bone marrow adipose tissue; *S_FF_*, signal fat fraction; WFI, chemical shift encoding-based water–fat imaging; mGRE, multiple gradient echoes; rBMAT, regulated BMAT; cBMAT, constitutive BMAT; ^1^H-MRS, single-voxel proton magnetic resonance spectroscopy; ROIs, regions of interest; CL, average fatty acid chain length; *ndb*, number of double bonds; *nmidb*, methylene-interrupted double bonds.
