# Supplementary figures and images for: Gender- and Age-Associated Differences in Bone Marrow Adipose Tissue and Bone Marrow Fat Unsaturation Throughout the Skeleton, Quantified Using Chemical Shift Encoding-Based Water–Fat MRI
Source: Front Endocrinol (Lausanne). 2022 Apr 27;13:815835. doi: 10.3389/fendo.2022.815835 (PMC9094426; doi:10.3389/fendo.2022.815835)

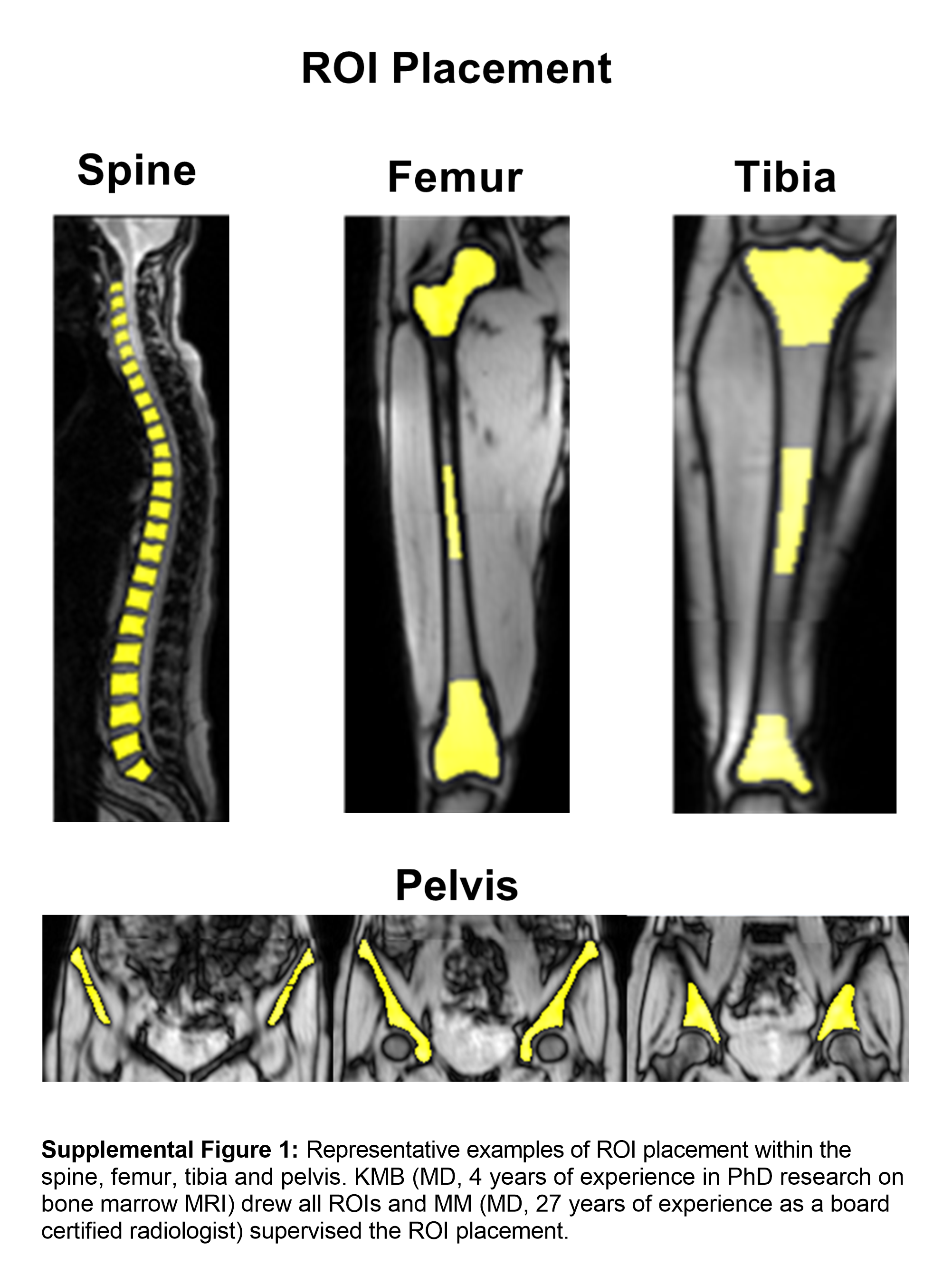

Supplement: Supplementary file 1 [file Image_1.tif]

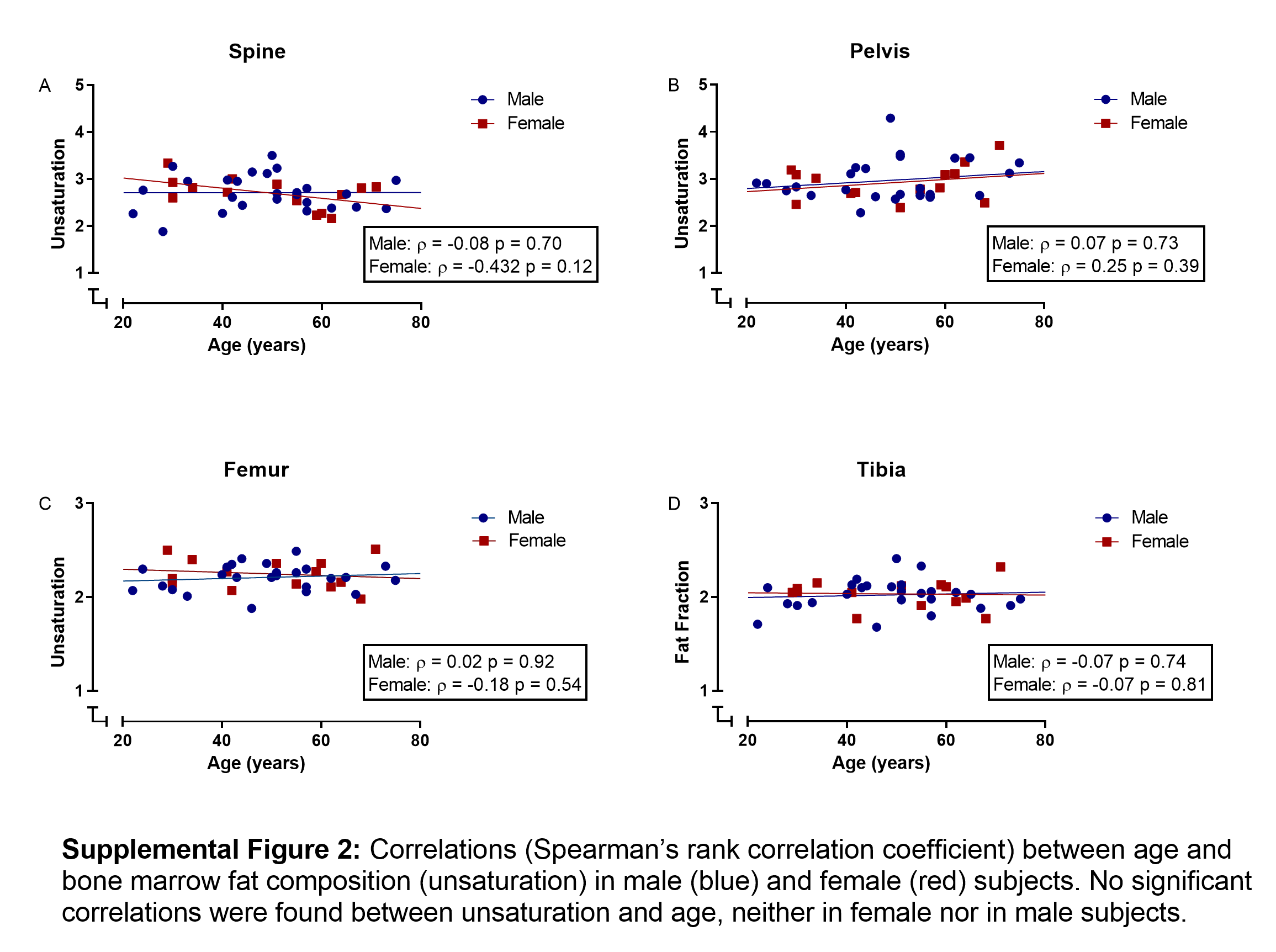

Supplement: Supplementary file 2 [file Image_2.tif]
